# Intracranial hemorrhage in a patient with severe COVID-19 acute respiratory distress syndrome on Veno-venous extra corporeal membrane oxygenation: A case report^☆^

**DOI:** 10.1016/j.amsu.2021.103033

**Published:** 2021-12-03

**Authors:** Anveshi Sathyavadhi, Anand Gupta, Vishnu Mahathi Avadhanam, Siva Kumar Reddy Lakkireddygari

**Affiliations:** Department of Critical Care Medicine, AIG Asian Institute of Gasteroenterology Hospitals, Gachibowli, Hyderabad, Telangana, India

**Keywords:** COVID −19, Intracranial hemorrhage, Veno-venous extra corporeal membrane oxygenation, Decompressive craniectomy, Case report

## Abstract

**Introduction and importance:**

COVID-19 can lead to severe acute respiratory distress syndrome (ARDS) where Veno-Venous Extra Corporeal Membrane Oxygenation (V–V ECMO) may be utilized for patients with severe respiratory failure. Our case report highlights a life threatening complication of V–V ECMO - intracranial hemorrhage (ICH), in a patient being treated for severe COVID-19 ARDS.

**Case presentation:**

A 41-year-old male of Indian ethnicity with no known comorbidities presented with an 8 day history of fever and dyspnoea. The patient was diagnosed with COVID-19 through a positive RT PCR test and his clinical condition progressively deteriorated requiring mechanical ventilation. Inspite of lung protective ventilation strategies and prone ventilation, there was no improvement in oxygenation. Therefore, the patient was placed on extra corporeal life support. On day three of V–V ECMO, the patient developed anisocoria and his GCS dropped to E1VTM1. A non-contrast CT brain scan revealed a large intraparenchymal hemorrhage in the right frontoparietal lobe with an extension into the right lateral and third ventricles leading to an emergency decompressive craniectomy with lax duroplasty.Post intracranial hemorrhage,ECMO support was continued without systemic anticoagulation. Despite a transient improvement in his GCS post surgery, the patient eventually succumbed to refractory septic shock with multi organ dysfunction syndrome.

**Clinical discussion and conclusion:**

Balancing anticoagulation therapy is one of the biggest challenges in managing ECMO support for COVID-19 ARDS. ICH is a rare and potentially fatal complication of V–V ECMO with an apparently higher incidence among COVID-19 patients. Neurosurgical procedures may be considered in such patients when no other possible management strategies are available (and the risk of death is imminent).

## Highlights

• Our case report highlights continuation of the ECMO run for 7 days without further complications and without systemic anticoagulation after the ICH was discovered.
• Neurosurgery was performed while the patient was on ECMO without anticoagulation. The cause of death of this patient was not due to the neurosurgical intervention itself, but due to septic shock one week later.
• Close monitoring of haematological parameters was done using thromboelastography (TEG), activated clotting time (ACT), activated partial thromboplastin time (aPTT).

## 1 Introduction

COVID-19 can lead to severe ARDS where V–V ECMO is utilized for patients with severe respiratory failure. Latest guidelines by ELSO (Extracorporeal Life Support Organization) concluded that the expected outcomes for COVID-19 patients are comparable to patients supported with V–V ECMO in the pre-pandemic era [1]. The incidence of central nervous system hemorrhage as a complication of ECMO in the ARDS cohort of COVID 19 patients as per the ELSO registry is 7% [2]. 10.13039/501100001282ICH is a well documented complication in adults on ECMO life support and is associated with high mortality. Evidence indicates that ICH is caused by both pre-ECMO morbidity and ECMO - induced disruption of coagulation. Management during ECMO requires striking a balance between pro-and anticoagulatory demands. Neurosurgical intervention can lead to severe morbidity but has been selectively successful in some cases [3]. We present a case report of a 41-year-old male with severe COVID-19 ARDS who developed intracranial hemorrhage on day 3 after initiation of V–V ECMO and subsequently underwent emergency neurosurgery. Globally, there are few documented case series of surgical intervention for ICH in patients on extra corporeal life support without systemic anticoagulation especially in COVID-19. To our knowledge, this is the first published case report of neurosurgery performed on a patient while on VV ECMO in India [4].

## 2 Case Presentation

A 41-year-old male of Indian ethnicity with no comorbidities presented with an 8 day history of fever and dyspnoea. His COVID-19 RT PCR test was positive. He did not have any significant past medical or surgical history. He had no known drug allergies. He was not on any medication at the time of presentation. His family history was not significant for any inheritable conditions. There was no history of alcohol consumption or smoking. He initially received treatment at a local hospital where his oxygen requirement at admission was 15 lit O_2_/min via a non-rebreathing mask. HRCT chest scan done on day 8 of illness showed CT severity score of 14/25. A repeat HRCT chest scan done on day 15 of illness showed a CT severity score of 20/25. Due to worsening hypoxia and tachycardia, CT pulmonary angiogram was done on day 20 of illness which was negative for pulmonary thromboembolism. His oxygen support was extended to intermittent non-invasive ventilation (NIV). On day 26 of illness, he developed severe respiratory distress and was placed on mechanical ventilator support.

At this stage, he was shifted to our hospital for extra corporeal life support. At the time of referral, he was on ventilator support with 90% FiO2 and a PEEP of 10. P/F ratio was 60 and driving pressures were 30 cms H_2_O. Acute Physiology and Chronic Health Evaluation (APACHE II) score was 14. He was maintained on prone ventilation for 16 hours. After the first cycle of proning, he continued to have persistent hypoxemia and carbon dioxide retention despite lung-protective ventilation. His Respiratory Extracorporeal Membrane Oxygenation Survival Prediction Score (RESP) was 3, with a predicted survival chance of 76%. A decision was made to initiate V–V ECMO (Maquet Rotaflow) on day 3 of mechanical ventilation. Cannulation was done with a double-lumen single 27F jugular venous cannula (paraglide) under transesophageal echocardiography guidance. Heparin infusion was started for anticoagulation. Monitoring of anticoagulation was done using activated clotting time (ACT) and activated partial thromboplastin time (aPTT) levels with a target range of 180–220 seconds for ACT and 60–80 seconds for aPTT. Mechanical ventilation settings were changed to ultra lung-protective ventilation. Driving pressures were adjusted to 15 cm H_2_O.

**Fig. 1:**
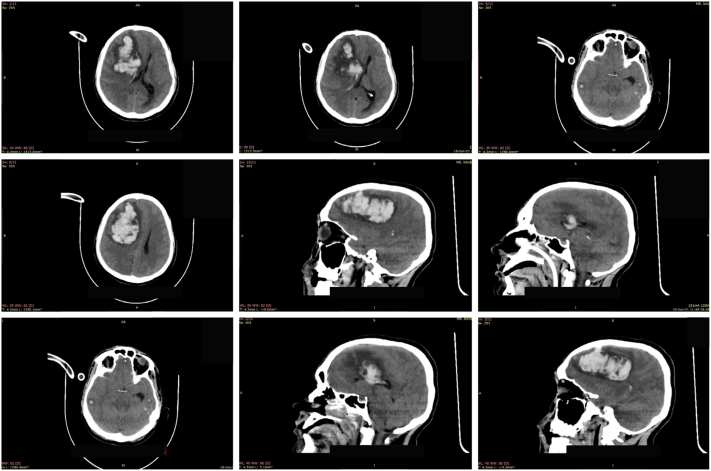
**Non-contrast CT brain scan** showing a large (8.7x5.7 × 4.2 cm) intraparenchymal hemorrhage seen in the right frontoparietal lobe with an extension of hemorrhage into the right lateral and 3rd ventricles, midline shift of 17 mm towards the left side, and effacement of suprasellar cisterns. In addition to focal hemorrhages in bilateral and temporal lobes, a small sulcal subarachnoid hemorrhage in the right temporoparietal lobe.

On day 3 of ECMO, his GCS was E1VTM1, and anisocoria was noted. A non-contrast CT brain scan revealed a large intraparenchymal hemorrhage (Fig. 1). The ECMO run continued hereafter without heparin and prophylactic levetiracetam was added to prevent seizures. ACT levels were noted to be in the range of 140–170 sec. A decision to proceed with decompressive craniectomy was taken by the neurosurgery team. After taking consent surgery was performed by the chief of neurosurgery under general anaesthesia at our hospital, which is a multi-specialty tertiary care center. Pre-operatively coagulation parameters included a platelet count of 1.5 lakh cells/mm3, ACT of 150 seconds, aPTT of 30 seconds, an INR of 1.2. Within 3 hours of diagnosis of the ICH, right frontotemporoparietal decompressive craniectomy was done with lax duroplasty.

On postoperative day 4 (day 8 of V–V ECMO), the patient showed spontaneous eye-opening and non-purposeful movements of the right upper limb (with a power of 3/5) and lower limb (with a power of 1/5). However, on day 9 of V–V ECMO, the patient developed septic shock. Blood cultures were positive for E.coli and multi-drug resistant *Klebsiella pneumoniae*. Antibiotic therapy was escalated as per culture and sensitivity reports and inotrope support was initiated. Despite aggressive invasive organ-supportive care the patient progressively deteriorated and eventually succumbed due to refractory septic shock with multi-organ dysfunction syndrome on day 12 of V–V ECMO.

## 3 Discussion

The strength of this case lies in the continuation of the ECMO run for 7 days without further complications and without systemic anticoagulation after the ICH was discovered. Neurosurgery was performed while the patient was on ECMO without anticoagulation. The cause of death of this patient was not due to the neurosurgical intervention itself, but due to septic shock one week later. The case was managed by a multidisciplinary team of intensivists, neurosurgeons, perfusionists, respiratory therapists and expert nursing staff. The limitation is that this is a single center observation limited to one case. Out of 5 V–V ECMO runs for severe COVID-19 ARDS at our center, 2 patients had ICH. One patient succumbed before intervention and the other patient is the subject of this case report. Therefore, our findings cannot be extrapolated to the general population.

The incidence of ICH during V–V ECMO for COVID-19 patients is reported to be higher when compared to non-COVID V–V ECMO runs. An early case series by Usman et al. reported 4 out of 10 COVID-19 patients (40%) with devastating intracranial hemorrhage on V–V ECMO. This was significantly higher than seen previously at the authors’ institution; where there was less than 1% intracranial hemorrhage during non-COVID lung rescue VV ECMO [5]. In another case series of 12 COVID-19 patients on V–V ECMO; Masur et al. reported intracranial hemorrhage among 5 of 12 patients (41.7%) [6].

Neurosurgical procedures in patients on anticoagulation carry a high risk of mortality. However, it may be considered in patients where no other possible management strategies are available and the risk of death is imminent [7]. Prinz et al. reported high mortality of ECMO-associated, space-occupying ICH requiring surgical evacuation; however, they pointed out that the detrimental effect of the surgical procedure is even less than the detrimental effect of the space-occupying hematoma itself. Their analysis of neurosurgical relevant ICHs showed an overall mortality rate as high as 80% [8]. Based on this, we made a decision to take our patient up for neurosurgery while on V–V ECMO. Post-surgery, we decided to continue ECMO support for our patient without anticoagulation and used thromboelastography (TEG) for monitoring.

Fletcher-Sandersjöö et al. described the possible pathophysiology of ICH in patients on V–V ECMO and proposed that it might involve an alteration in hemostasis as a likely significant mechanism. ECMO support itself results in thrombocytopenia, factor XIII deficiency, acquired von Willebrand syndrome, fibrinogen deficiency, and pump-induced platelet dysfunction. However, we did not observe most of these findings in our patient. They also discussed pre-ECMO factors that may also play a role in ICH development. In V–V ECMO, abrupt CO2, or O2 changes at ECMO cannulation can disrupt cerebral perfusion, which is further decreased by the use of potent sedatives, and has been linked to cerebral desaturation during ECMO initiation, as well as impairing cerebral autoregulation which in turn can precede ischemic stroke leading to ICH [3].

The determinants of mortality in patients with ICH on V–V ECMO as per Prinz et al. confirmed the independent effect of a larger ICH volume, coagulation that is not normalized within 12 hours after ICH onset, presence of intraventricular hemorrhage (IVH), larger surgical intervention, and a fluid level inside the ICH.^7^ Our patient had 3 out of the above 5 parameters - large ICH volume (175 ml), larger surgical intervention (decompressive hemicraniectomy) and presence of IVH. As per another study, the factors influencing 30-day mortality in patients with ICH while on V–V ECMO included - a significantly low level of consciousness at ICH diagnosis, presence of intraparenchymal hemorrhage (IPH), large IPH volume (>38 ml), presence of IVH, Subarachnoid Hemorrhage (SAH) Fisher grade [5], hydrocephalus, midline shift (0–11 mm) and absent basal cisterns.^6^ Our patient fulfilled 6 of the above 8 parameters. The unfavorable outcome of our patient is in line with the findings of previous studies. The lack of change in ECMO anticoagulation regimens might be one of the reasons behind the stagnation in rates of ICH occurrence and mortality [9].

## 4 Conclusion

Our case report highlights that neurosurgical interventions can be safely performed on patients on extra corporeal life support although there is a risk of mortality. The detrimental effects of the surgery are lower than the detrimental effects of the ICH. Extra corporeal life support can be run without anticoagulation in patients who have severe active bleeding or at high risk of bleeding. This case adds to the evolving experience of the challenges faced in balancing anticoagulation therapy during extra corporeal life support. Close monitoring of all hematologic parameters is recommended during ECMO support along with a low threshold for neuroimaging. Larger, multicenter studies are warranted to evaluate the long-term feasibility of ECMO without systemic anticoagulation.

## Sources of funding

This research did not receive any specific grant from funding agencies in the public, commercial, or not-for-profit sectors.

## Ethical approval

Institutional Ethical Committee approved - Asian Institute of Gastroenterology, Gachibowli, Hyderabad, Telangana, India.

Approval number: AIG/IEC - post CT50/07.2021-01

## Consent

Written informed consent was obtained from the patient for publication of this case report and accompanying images. A copy of the written consent is available for review by the Editor-in-Chief of this journal on request.

## Author contribution

All authors have contributed equally. All authors read and approved the final manuscript.

## Research registration

Not applicable.

## Guarantor

Dr. Anveshi Sathyavadhi.

## Provenance and peer review

Not commissioned, externally peer-reviewed.

## Declaration of competing interest

The authors declare no conflict of interest.

## List of abbreviations

RT PCR: Reverse Transcription Polymerase Chain Reaction
GCS: Glasgow Coma Scale
CT: Computed Tomography
HRCT: High Resolution Computed Tomography
P/F ratio: PaO2/ FiO2 ratio
PEEP: Positive End Expiratory Pressure
ICH: Intracranial hemorrhage
V–V ECMO: Venovenous Extracorporeal membrane oxygenation
COVID-19: Coronavirus disease 2019
